# Development of Sensitive and Specific Analysis of Vildagliptin in Pharmaceutical Formulation by Gas Chromatography-Mass Spectrometry

**DOI:** 10.1155/2015/707414

**Published:** 2015-11-22

**Authors:** Ebru Uçaktürk

**Affiliations:** Faculty of Pharmacy, Department of Analytical Chemistry, Hacettepe University, Sıhhıye, 06100 Ankara, Turkey

## Abstract

A sensitive and selective gas chromatography-mass spectrometry (GC-MS) method was developed and fully validated for the determination of vildagliptin (VIL) in pharmaceutical formulation. Prior to GC-MS analysis, VIL was efficiently derivatized with MSTFA/NH_4_I/*β*-mercaptoethanol at 60°C for 30 min. The obtained O-TMS derivative of VIL was detected by selected ion monitoring mode using the diagnostic ions* m/z* 223 and 252. Nandrolone was chosen as internal standard. The GC-MS method was fully validated by the following validation parameters: limit of detection (LOD) and quantitation (LOQ), linearity, precision, accuracy, specificity, stability, robustness, and ruggedness. LOD and LOQ were found to be 1.5 and 3.5 ng mL^−1^, respectively. The GC-MS method is linear in the range of 3.5–300 ng mL^−1^. The intra- and interday precision values were less than ≤3.62%. The intra- and interday accuracy values were found in the range of −0.26–2.06%. Finally, the GC-MS method was successfully applied to determine VIL in pharmaceutical formulation.

## 1. Introduction

Vildagliptin (VIL) [(S)-1-[N-(3-hydroxy-1-adamantyl)glycyl]pyrrolidine-2-carbonitrile] is a new oral antidiabetic drug belonging to the class of dipeptidyl peptidase-4 inhibitor and is used as treatment of type 2 diabetes. It is given alone or in combination therapy with metformin, sulfonylurea, or thiazolidinedione [1].

VIL (trade names: Galvus, Jalra, and Xiliarx) is approved in many countries worldwide and also a fixed-dose combination with metformin (trade names: Eucreas, Icandra, and Zomarist) is available in the pharmaceutical market [2, 3].

Up to date, analysis of VIL alone and together with metformin in pharmaceutical formulations was performed by high performance liquid chromatography (HPLC) and capillary electrophoresis (CE) coupled with ultraviolet (UV) or photodiode array (PDA) detector [4–10]. Specificity of CE and some of HPLC methods was checked using the retention time confirmation and the spectral peak purity of VIL [4, 5]. VIL gives weak UV absorbance at a maximum wavelength 207 nm. Therefore, the reported analytical methods have low sensitivity and selectivity.

HPLC is mostly preferred in the quality control of pharmaceutical formulations because of its simplicity, cost effectiveness, and being accessible in most laboratories. Gas chromatography-mass spectrometry (GC-MS) is mostly employed to analyze volatile drugs and residual solvents and to analyze some weak compounds or lack of chromophore groups. GC-MS gives many advantages over HPLC such as high efficiency, sensitivity, specificity, short analysis time, and small sample volume.

In this study we proposed development of a selective, sensitive, and fully validated GC-MS method for the analysis of VIL in pharmaceutical formulation. Herein, VIL was derivatized before GC-MS analysis. Silylation reaction was used for derivatization. Silylation reaction was optimized investigating the following parameters: catalyst, derivatization time, and temperature [11, 12]. Then, the optimized GC-MS method was fully validated. The developed and validated GC-MS method was applied to determine VIL in tablet formulation. This proposed method is important because of the first reported GC-MS method.

## 2. Experimental Part

### 2.1. Chemicals

VIL was obtained from Central Institute of Hygiene of Turkey. Nandrolone (IS) was kindly provided from Turkish Doping Control Center. Metformin, N-methyl-N-(trimethylsilyl)trifluoroacetamide (MSTFA), *β*-mercaptoethanol, and ammonium iodide (NH_4_I) were supplied from Sigma-Aldrich. All other chemicals were of analytical reagent grade.

### 2.2. Instrumentation and GC-MS Conditions

GC-MS analysis was performed on a 6890 N Agilent GC coupled with 5973 N mass selective detector. 5% phenyl methylpolysiloxane capillary column (30 m × 0.25 mm i.d. with 0.25 *µ*m film thickness, Agilent Technologies, USA) was used for chromatographic analysis. Injection was carried out in split mode with split ratio of 10 : 1 and 1 *µ*L of sample was injected to capillary column. The oven temperature was programmed as follows. The initial temperature was set at 200°C and then the temperature was increased to 300°C at a rate of 25°C min^−1^, and it was held at 300°C for 2 min. The total run time is 6 min. The MS was operated in electron impact ionization mode. Selected ion monitoring mode was performed to monitor VIL and IS. The identification masses were* m/z* 223 and 252 for O-TMS derivatives of VIL. The ions* m/z* 223 for VIL and 418 for IS were the most abundant ions. Thus, these ions were selected for quantitation. The temperatures of front inlet, ion source, and interface were 280, 230, and 280°C, respectively.

### 2.3. Preparation of Standard Solutions

Stock solution of VIL (500 ng mL^−1^) was prepared by dissolving the VIL in methanol : water (50 : 50, v : v). In order to prepare standard stock solution of IS (1000 ng mL^−1^), appropriate amount of IS was dissolved in methanol. Working solutions of VIL and IS (10 ng mL^−1^) were prepared by serial dilution of the stock solutions with methanol. Stock and working solutions were kept at 4°C for one month.

### 2.4. Preparation of Derivatization Reagents

In order to prepare the stock solution of MSTFA/NH_4_I/*β*-mercaptoethanol (100 : 2 : 6, v : w : v), 100 mg of NH_4_I and 300 *µ*L of *β*-mercaptoethanol were added to 5 mL of MSTFA. This stock solution was then diluted to desired concentration (1000 : 2 : 6, v : w : v) with MSTFA. Solutions were stored at 4°C in the dark.

### 2.5. Preparation of Trimethylsilyl (TMS) Ether Derivatives of VIL and IS

To prepare the TMS ether derivatives of VIL and IS, 40 *µ*L of derivatization reagent (MSTFA or MSTFA/NH_4_I/*β*-mercaptoethanol mixture) was added to dry residue containing VIL. This solution was left at 60°C for 30 min in a heating block.

### 2.6. Preparation of the Placebo Samples

The specificity of the GC-MS method was shown to test the placebo tablet containing lactose anhydrous, magnesium stearate, cellulose microcrystalline, and sodium starch glycolate. In order to prepare placebo tablet, the excipients were first taken into 100 mL flask and 80 mL of methanol : water (1 : 1, v : v) was added into the flask. The solution was left in the ultrasonic bath for 15 min. Then, it was completed to 100 mL with mixture of methanol : water (1 : 1, v : v). Finally, it was filtrated by 0.22 *µ*m membrane filter.

### 2.7. Method Validation

In order to show performance of the developed GC-MS method, the GC-MS method was validated by investigating the following parameters: specificity, limit of detection (LOD) and quantification (LOQ), linearity, precision, accuracy, stability, robustness, and ruggedness.

#### 2.7.1. Specificity Test

The GC-MS method was tested if there are any interferences because of the excipients in the pharmaceutical formulation containing VIL active ingredient. Thus, placebo of tablet was prepared as described in Section 2.6 and was analyzed by the GC-MS method. In addition, to evaluate the interference of metformin commonly used medication, metformin standard solution was derivatized as described in Section 2.5 and analyzed by GC-MS. The diagnostic ions for VIL (*m/z* 223 and 252) and IS (*m/z* 418) were extracted in the chromatograms obtained from placebo sample and metformin.

#### 2.7.2. Limits of the GC-MS Method and Linearity

LOD and LOQ were determined by signal to noise ratio (S/N) for the two most intense diagnostic ions (*m/z* 223 and 252). Precision and accuracy at LOQ level were also evaluated. Linearity was investigated in the six concentration levels, 3.5 (LOQ), 10, 50, 100, 200, and 300 ng mL^−1^. Calibration curve was plotted by peak area ratio of VIL to IS versus the concentration of VIL.

#### 2.7.3. Precision and Accuracy

Precision and accuracy were evaluated as intra- and interday. The intraday accuracy and precision were determined by analyzing the standard solutions of VIL prepared in six independent series at three concentration levels (15, 150, and 250 ng mL^−1^) and these solutions were analysed on the same day.

In the interday accuracy and precision, standard solutions of VIL at three concentration levels (15, 150, and 250 ng mL^−1^) were prepared for six different days and the solutions were analysed each day. Injection repeatability of the GC-MS method was also performed by injecting ten times of the standard VIL solution at the concentration of 150 ng mL^−1^. Precision and accuracy were expressed by percent relative standard deviation (RSD) and bias, respectively.

#### 2.7.4. Stability

The stability of the standard stock solutions of VIL (500 ng mL^−1^) and stability of IS (1000 ng mL^−1^) were tested at +4°C for 30 days. In addition, in order to examine the autosampler stability of O-TMS derivative of VIL, O-TMS derivative at three concentration levels (15, 150, and 250 ng mL^−1^) was placed in autosampler for 12 hours.

After the storage, the standard stock solution of VIL and IS and the O-TMS derivative kept in autosampler for 12 hours were analyzed by GC-MS. The results were compared with the results obtained from freshly prepared solutions.

#### 2.7.5. Robustness and Ruggedness

The robustness and ruggedness of the developed method were evaluated by Plackett-Burman experimental design. Selected parameters, levels, and designed experimental matrix were shown in Tables 1 and 2. The peak area ratio of VIL to IS was chosen as response.

### 2.8. Determination of Vildagliptin in Pharmaceutical Tablet Formulation

Applicability of the developed GC-MS method was assessed by analyzing the tablet formulation containing VIL active ingredient (GALVUS/50 mg VIL/tablet). Ten of the tablets were homogenized in a mortar. An amount of 50 mg was transferred into the 100 mL volumetric flask. 80 mL of methanol : water (1 : 1, v : v) was then added to the volumetric flask and left in the ultrasonic bath for 15 min. The solution was made up to the 100 mL with methanol : water (1 : 1 v : v). Finally, it was filtrated by 0.22 *µ*m membrane filter.

## 3. Results and Discussion

VIL does not contain any strong chromophore group. Therefore, it needs to be analyzed HPLC after post- or precolumn derivatization or using MS detectors. Thus, sensitive and specific analysis of VIL would be achieved.

**Figure 1:**
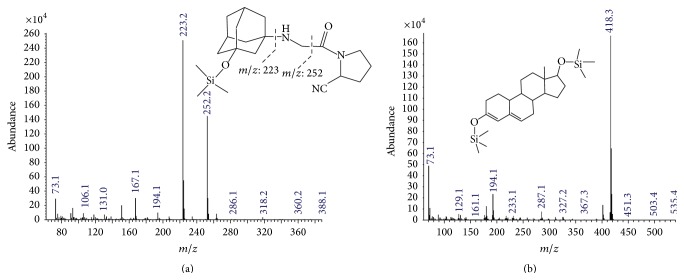
Mass spectrum of the O-TMS derivative of VIL (a) and bis-OTMS derivative of IS (b).

In our study, we aimed to develop a GC-MS method for its analysis. VIL was derivatized prior to GC-MS analysis. Thus, it has thermal stability and/or enough volatility. VIL contains hydroxyl group and –NH group to be derivatized. In order to derivatize VIL, silylation reaction was tried. MSTFA is used as derivatization reagent. The results showed that only free hydroxyl group of VIL was derivatized and its O-TMS derivative was composed. It is believed that the –NH group of VIL is sterically hindered so that the silylation reaction is not possible in that side of the molecule. The mass spectrum and fragmentation of O-TMS derivatives of VIL were given in Figure 1.

In order to optimize derivatization reaction, parameters such as catalyst, derivatization time, and temperature were investigated. In the optimization studies, the quantitation ion of VIL (*m/z* 223) was monitored and the peak area value of the O-TMS derivative was compared.

**Figure 2:**
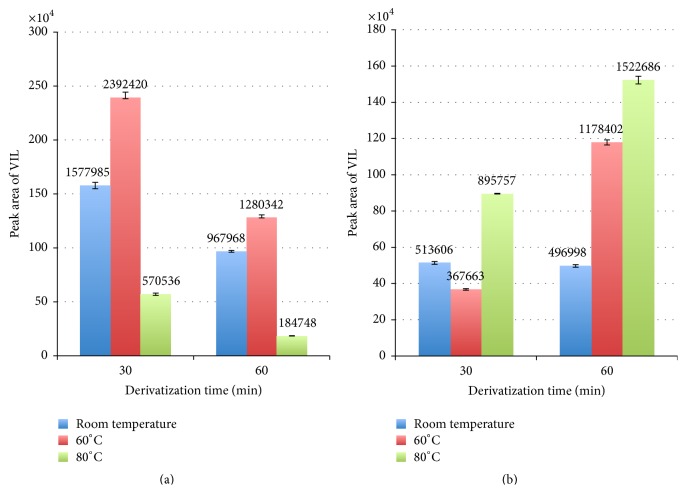
Yield of the derivatization reaction using MSTFA/NH4I/*β*-mercaptoethanol (a) and MSTFA (b) under different temperature and time periods.

In this study, MSTFA was used as silylation reagent and a mixture of NH_4_I/*β*-mercaptoethanol was added as base catalyst to increase the yield of the O-TMS derivative. The silylation reaction was performed using MSTFA alone and MSTFA/NH_4_I/*β*-mercaptoethanol with different temperature (room temperature, 60 and 80°C) and time intervals (30 and 60 min). The results show that using NH_4_I/*β*-mercaptoethanol as base catalyst increases the yield of the O-TMS derivatives of VIL (Figure 2). When the results were compared, the best result was taken at 60°C for 30 min.

Nandrolone was selected as IS. It was efficiently derivatized under the optimized derivatization conditions of VIL. The mass spectrometry of IS was given in Figure 1.

### 3.1. The GC-MS Method Validation

#### 3.1.1. Specificity Test

**Figure 3:**
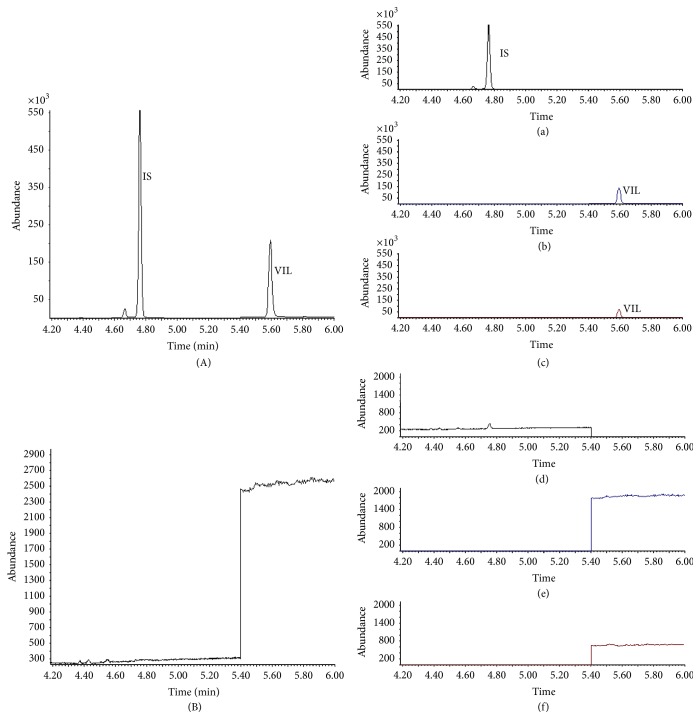
(A) Total ion chromatogram of VIL (100 ng mL^−1^) and IS. Selected ion chromatograms for* m/z* 418 (a), 223 (b), and 252 (c) belonging to standard solution containing VIL (100 ng mL^−1^) and IS. (B) Total ion chromatogram of the placebo tablet. Selected ion chromatograms for* m/z* 418 (d), 223 (e), and 252 (f) belonging to placebo tablet.

The chromatograms obtained from placebo tablet and metformin standard solution were examined at the retention time of VIL and IS by monitoring the ions of* m/z* 223, 252 (diagnostic ions for VIL), and* m/z* 418 (diagnostic ion for IS). Any interference because of the excipients and metformin was not observed indicating that the GC-MS method is specific (Figure 3).

#### 3.1.2. Limits of the Method (LOQ and LOD) and Linearity

LOD (S/G: 3) and LOQ (S/G: 10) were determined to be 1.5 ng mL^−1^ and 3.5 ng mL^−1^, respectively. Precision and accuracy at LOQ level were found to be 4.03% and −1.42%, respectively. The calibration curve ranging 3.5–300 ng mL^−1^ had a coefficient of determination (*r* ^2^) value (0.9968 ± 0.0008) greater than 0.99 indicating linear fit over the examined concentration range.

#### 3.1.3. Precision and Accuracy

The results of the intraday accuracy and precision were summarized in Table 3. Values of precision did not exceed 3.62% and accuracy ranged from −0.26 to 2.06%. Injection repeatability of the GC-MS method was calculated as 1.26%.

#### 3.1.4. Stability

The stability results of the standard stock solutions of VIL (500 ng mL^−1^) and IS (1000 ng mL^−1^) show that there is no significant difference between the storage periods. Therefore, the standard solutions of VIL and IS were prepared monthly in the study. In addition, it was found that the O-TMS derivative of VIL was stabile in autosampler for 12 hours.

#### 3.1.5. Robustness and Ruggedness

**Figure 4:**
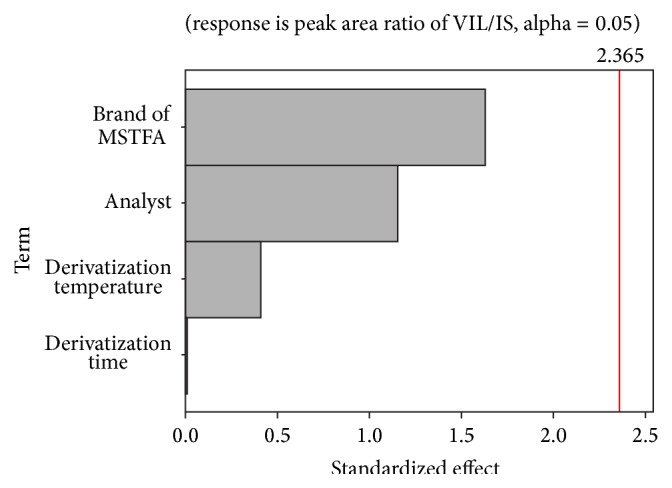
Pareto chart of the standardized effect.

In order to evaluate the robustness and ruggedness of the developed method, ANOVA test was used. Thus, the effects of each parameter on the response (peak area ratio of VIL to IS) were determined. The normal probability plot of the standardized effects was shown in Figure 4. The results showed that the effects of selected parameters on the developed method were not significant (*p* = 0.228 > 0.05 for analyst, *p* = 0.147 > 0.05 for brand of MSTFA, *p* = 0.996 > 0.05 for derivatization time, and *p* = 0.697 > 0.05 for derivatization temperature).

## 4. Determination of Vildagliptin in Pharmaceutical Formulations

In order to show the reliability of the GC-MS method, the developed method was applied to determine VIL in tablet formulation (Galvus 50 mg/Tablet). Sample preparation was described in Sections 2.5 and 2.8 and then these samples were analyzed by GC-MS method. The content of the tablet was calculated from the calibration curve. The amount of VIL in tablet was found to be 50.12 ± 0.35 (mean ± S.E., 1.71% RSD) indicating that the proposed GC-MS method was reliable for quantitation of VIL in tablet.

## 5. Conclusion

In this study, we reported a GC-MS method for the determination of VIL for the first time. VIL was efficiently derivatized with MSTFA/NH_4_I/*β*-mercaptoethanol and converted into the O-TMS derivative. Derivatization procedure is simple and one-step. The total analysis time takes 6 min offering the high throughput analysis of VIL. The ions* m/z* 223 and 252 can be effectively used as diagnostic ions for the determination of VIL. Thus, the proposed GC-MS method would be much more specific and sensitive than the other reported methods using UV or PDA detection [4–10]. The developed GC-MS method can be considered sensitive, selective, precise, accurate, robust, and rugged according to the validation studies. The method was suitably used for determination of VIL in tablet formulation. This GC-MS method may be used for the routine determination of VIL in biological samples.

## Figures and Tables

**Table 1:** Selected method parameters and their levels for Plackett-Burman experimental design.

Selected variables	Low level (−)	High level (+)
Analyst	Analyst I	Analyst II
Brand of MSTFA	MSTFA I	MSTFA II
Derivatization time (min) (±5)	25	35
Derivatization temperature (°C) (±5)	55	65

**Table 2:** Conducted experimental runs for robustness and ruggedness studies.

Experiment number	Selected internal/external variables			
	Analyst	Brand of MSTFA	Derivatization time (min)	Derivatization temperature (°C)
1	+^a^	+	−^b^	+
2	−	+	+	−
3	+	−	+	+
4	−	+	−	+
5	−	−	+	−
6	−	−	−	+
7	+	−	−	−
8	+	+	−	−
9	+	+	+	−
10	−	+	+	+
11	+	−	+	+
12	−	−	−	−

^a^High level. ^b^Low level.

**Table 3:** Intra- and interday precision and accuracy results for VIL (*n* = 6).

Nominal amount (ng mL^−1^)	Interday			Intraday		
	*X* ± SE^a^	Precision^b^ (%)	Accuracy^c^ (%)	*X* ± SE^a^	Precision^b^ (%)	Accuracy^c^ (%)
15	15.31 ± 0.19	3.62	2.06	15.2 ± 0.22	3.04	1.33
150	150.7 ± 1.68	2.73	0.46	151.16 ± 1.49	2.41	0.77
250	249.3 ± 1.87	1.84	−0.26	251.01 ± 1.20	1.17	0.40

^a^ *X*  ± SE: mean ± standard error.

^b^Percent relative standard deviation.

^c^Bias: [(found − added)/added] × 100.
